# Creating Polyploid *Escherichia Coli* and Its Application in Efficient L‐Threonine Production

**DOI:** 10.1002/advs.202302417

**Published:** 2023-09-25

**Authors:** Sumeng Wang, Xuanmu Chen, Xin Jin, Fei Gu, Wei Jiang, Qingsheng Qi, Quanfeng Liang

**Affiliations:** ^1^ State Key Laboratory of Microbial Technology Shandong University Qingdao 266237 P. R. China; ^2^ Research Center of Basic Medicine Central Hospital Affiliated to Shandong First Medical University Jinan 250013 P. R. China

**Keywords:** cell division, L‐threonine production, metabolic engineering, polyploid *Escherichia coli*, synthetic biology

## Abstract

Prokaryotic genomes are generally organized in haploid. In synthetic biological research, efficient chassis cells must be constructed to produce bio‐based products. Here, the essential division of the *ftsZ* gene to create functional polyploid *E. coli* is regulated. The artificial polyploid *E. coli* containing 2–4 chromosomes is confirmed through PCR amplification, terminator localization, and flow cytometry. The polyploid *E. coli* exhibits a larger cell size, and its low pH tolerance and acetate resistance are stronger than those of haploid *E. coli*. Transcriptome analysis shows that the genes of the cell's main functional pathways are significantly upregulated in the polyploid *E. coli*. These advantages of the polyploid *E. coli* results in the highest reported L‐threonine yield (160.3 g L^−1^) in fed‐batch fermentation to date. In summary, an easy and convenient method for constructing polyploid *E. coli* and demonstrated its application in L‐threonine production is developed. This work provides a new approach for creating an excellent host strain for biochemical production and studying the evolution of prokaryotes and their chromosome functions.

## Graphical abstract

A polyploid *Escherichia coli* is created by regulating *ftsZ* expression level, which exhibits a highly stable chromosome, larger cell size, stronger low pH and acetate tolerance. The genes of the cell's main functional pathways are significantly upregulated in the polyploid *E. coli*. This polyploid *E. coli* shows great potential for L‐threonine production with the highest titer of 160.3 g L^−1^.

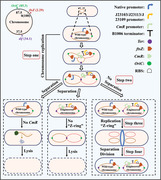

## 1 Introduction

The cores of synthetic biology are to construct and implement new genetic systems and reshape natural biological systems. Advances in synthetic biology highlight a wide range of bio‐based applications.^[^ ^1^ ^]^ Designing an efficient microbial cell for high‐value‐added chemical production is a current research focus in synthetic biology. The evolutionary trend is from haploid to polyploid cells, which have advantages over haploid cells.^[^ ^2^ ^]^ Polyploidy is a stable genetic state in which cells have more than two complete chromosomes, and the genomic content of such cells is multiplied.^[^ ^3^ ^]^ Natural polyploidy is common in eukaryotes such as plants, animals, and some yeasts. Studies show that polyploidy also occurs in some bacteria and archaea such as *Azotobacter vinelandii*, *Deinococcus radiodurans*, and *Epulopiscium*.^[^ ^2^, ^4^ ^]^ Although polyploidy remains underexplored in many contexts, and its effects in biological processes and phylogenesis are unclear, its genetic diversity is greatly enhanced owing to increased genome numbers, which improve cells’ resistance to environmental stressors.^[^ ^5^ ^]^ Gene redundancy in polyploid cells can prevent harmful mutations that might escape and persist in other genomes, allowing cells to survive robustly.^[^ ^3a^ ^]^ Additionally, the larger genome alters global transcription, metabolism levels and gene expression.^[^ ^6^ ^]^

As a haploid model organism, *E. coli* has a clear genetic background and fast growth rate and is easily genetically manipulated. It is widely used in genetics and microbial physiology research and is the most commonly used bacterial platform organism in industrial applications.^[^ ^7^ ^]^ Thus, constructing an artificial polyploid *E. coli* may enable building an efficient chassis cell for producing bio‐based products and analyzing the impact of adding chromosomes to prokaryotes. In eukaryotic cells, abnormal division increases the number of chromosomes,^[^ ^8^ ^]^ thus providing an opportunity to construct polyploid *E. coli* by controlling cell division. *ftsZ* is the main gene controlling cell division. Weak expression of *ftsZ* below its threshold can prevent cell division, thus preventing a new round of chromosome replication while still allowing ongoing replication to complete.^[^ ^9^ ^]^ During this time, cells grow filaments and contain increased numbers of improperly segregated chromosomes that extend along the cell filament. However, prolonged inhibition of division leads to quiescence, and the filamentous cells are finally lysed due to terminal cell‐cycle arrest. Conversely, high *ftsZ* expression allows cells to survive with normal division and chromosome segregation.^[^ ^10^ ^]^ Therefore, it may be possible to construct polyploid *E. coli* by adjusting *ftsZ* expression levels to an appropriate concentration.

L‐threonine (CAS: 72‐19‐5) is one of the essential amino acids; it belongs to the aspartic acid family of amino acids. It is used widely in food, pharmaceuticals, and cosmetics. The global production of L‐threonine has reached ≈700 000 metric tons annually, so it is one of the four major commercial amino acids.^[^ ^11^ ^]^ L‐threonine is mainly produced by microbial fermentation, using oxaloacetic acid or fumaric acid as the precursor, through six steps respectively catalyzed by aspartate aminotransferase (encoded by *aspC*), aspartate kinase (encoded by *thrA*, *metL*, or *lysC*), aspartate semialdehyde dehydrogenase (encoded by *asd*), homoserine dehydrogenase (encoded by *thrA* or *metL*), homoserine kinase (encoded by *thrA* or *metL*), and L‐threonine synthase (encoded by *thrC*).^[^ ^12^ ^]^ Common strategies for improving microbial L‐threonine production include enhancement of the L‐threonine synthesis pathway, weakening of competing pathways, enhancement of L‐threonine transport, enhancement of cofactor supply, and removal of feedback inhibition.^[^ ^11^, ^13^ ^]^ Previous studies have reported that regulating *ftsZ* expression can increase cell volume to enhance the production of inclusions such as polyhydroxyalkanoates, and can decrease cell volume to enhance cell robustness to improve the production of toxic chemicals such as isobutanol.^[^ ^14^ ^]^ However, the effect of regulating *ftsZ* expression on common products, such as L‐threonine, has not been reported.

This study was conducted to design a simple and accessible method for creating polyploid *E. coli* and explore its contribution in synthesizing high‐value‐added chemicals. We created polyploid *E. coli* containing 2–4 chromosomes by regulating *ftsZ*, a gene essential for division, with low pH tolerance, acetate resistance and enhanced metabolic pathways. This polyploid *E. coli* is highly stable and can increase L‐threonine production.

## 2 Results

### 2.1 Polyploid *E. coli* Design

**Figure 1:**
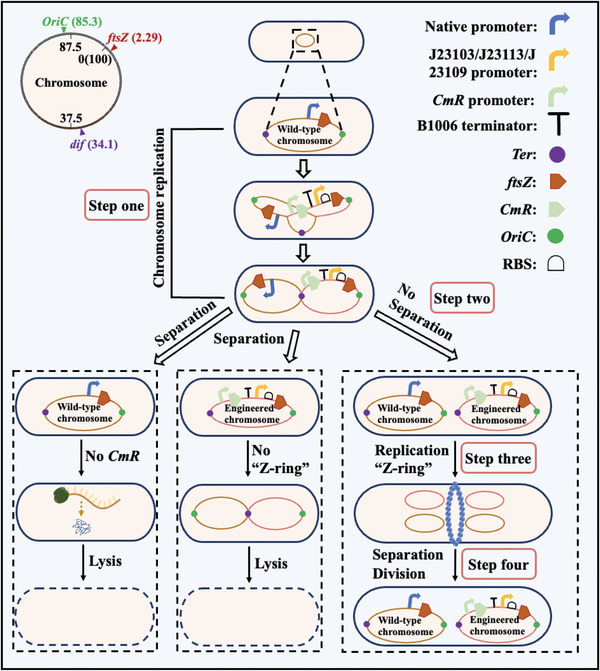
Schematic of the design process for polyploid *E. coli*. Polyploid *E. coli* was constructed via weak *ftsZ* expression. The regulation cassette containing *CmR*‐terminator‐promoter‐RBS was inserted before the start codon of *ftsZ* in a replication fork during replication. Cells containing wild‐type or engineered chromosomes cannot survive under chloramphenicol supplementation. On the *E. coli* chromosome map (0–100 min), a distance of 5 min equals ≈230 kb of DNA.

A weak *ftsZ* expression cassette containing chloramphenicol acetyltransferase (*CmR*) was inserted into one of the replication forks formed during *E. coli* replication to ensure chromosome multiplication and cell division for survival. Polyploid *E. coli* containing both wild‐type and engineered chromosomes was constructed under screening pressure from chloramphenicol and weakly expressed *ftsZ*. The detailed design of this study is as follows (**Figure** **1**). On the *E. coli* chromosome map, *ftsZ* was located at 2.29 min, near *OriC* and away from the terminator. In step one, during *E. coli* replication, a weak expression cassette containing *CmR*‐terminator‐promoter‐ribosome binding site (RBS) was inserted in front of the ATG start codon of *ftsZ*. The expression cassette could be integrated into one or two replication forks before *ftsZ* during chromosome replication. However, inserting only one replication fork ensures that cells survive using the other replication fork containing wild‐type *ftsZ* to express *ftsZ* normally. In step two, in the presence of chloramphenicol, *E. coli* containing the wild‐type chromosome without *CmR* cannot survive (Figure 1). The FtsZ‐based divisome regulates *E. coli* division. FtsZ is a tubulin homolog that forms a “Z ‐ring” to recruit other division proteins and divide cells in two.^[^ ^15^ ^]^ Therefore, *E. coli* containing the engineered chromosome ultimately fails to grow because of low FtsZ expression. Only *E. coli* containing both the no separated wild‐type and engineered chromosomes can grow normally. In step three, the two chromosomes in the cell perform the next replication cycle. With the accumulation of FtsZ in the wild‐type chromosomes, a Z‐ring forms in the middle of the cell. In step four, replicated chromosomes segregate and the cell divides into polyploid daughter cells. These cells use the wild‐type chromosome to normally express *ftsZ* to regulate cell division and use the engineered chromosome to express *CmR* to resist chloramphenicol pressure.

**Figure 2:**
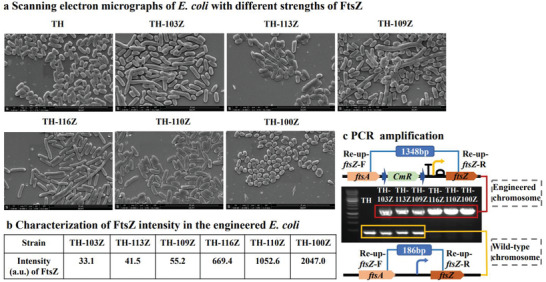
Effects of different FtsZ intensities on cell morphology and chromosome numbers. a) Scanning electron micrographs of *E. coli* with different FtsZ strengths. Scale bar, 10 µm. b) Fluorescence intensity values of expression elements of J23103/J23113/J23109‐B0033 and J23116/J23110/J23100‐B0034 were normalized to cell density (OD_600_) and used to control *ftsZ* expression in strains TH‐103Z, TH‐113Z, TH‐109Z, TH‐116Z, TH‐110Z, and TH‐100Z, respectively. c) Chromosome numbers of cells with different FtsZ strengths were analyzed via PCR amplification with primers Re‐up‐*ftsZ*‐F/Re‐up‐*ftsZ*‐R upstream and downstream of the integrated expression cassette *CmR*‐terminator‐promoter‐RBS.

Because *ftsZ* promoters are located in the intragenic region of essential gene *ftsA*, *ftsZ* promoters cannot be deleted. To avoid the influence of *ftsA* expression, the *CmR*‐terminator‐promoter‐RBS expression cassette was inserted before the ATG start codon of *ftsZ* when *E. coli* grew at OD_600_ 0.6 (Figure 1). We selected B1006 terminator with 99% transcriptional termination efficiency and various promoter strengths and RBS from the iGEM library. Results based on reporter gene characterization showed intensities of 33.1, 41.5, and 55.2 a.u. for promoters J23103, J23113, J23109 and RBS B0033 in combination, respectively. The intensities for promoters J23116, J23110, J23100 and RBS B0034 in combination were 669.4, 1052.6, and 2047.0 a. u., respectively (**Figure** **2**b). We observed a similar trend in *ftsZ* expression for these combinations of promoters/RBS when using western blotting as we did when using reporter genes (Figure S1, Supporting Information). As the *ftsZ* expression intensity increased from 669.4 arbitrary units (a.u.) in strain TH‐116Z to 2047.0 a.u. in strain TH‐100Z, the *E. coli* changed shape from filamentous to oval (Figure 2a,b). Weak and strong *ftsZ* expression reportedly lead to filamentous and oval *E. coli* growth, respectively.^[^ ^16^ ^]^ However, when *ftsZ* expression was further weakened in strains TH‐103Z, TH‐113Z, and TH‐109Z, the cells grew at inconsistent lengths and were slightly longer than normal *E. coli* owing to the additional chromosomes.^[^ ^3b^ ^]^ Single colonies were isolated under chloramphenicol and amplified using primers Re‐up‐*ftsZ*‐F and Re‐up‐*ftsZ*‐R, which were designed at intragenic regions of *ftsA* and *ftsZ* (Figure 2c). Strains TH‐103Z, TH‐113Z and TH‐109Z were amplified with two DNA fragments: 186 bp of the wild‐type chromosome and 1348 bp of the engineered chromosome. However, the other strains were amplified only with a 1348‐bp DNA fragment of the engineered chromosome (Figure 2c). Thus, strains TH‐103Z, TH‐113Z and TH‐109Z likely contained both wild‐type and engineered chromosomes. The strongly *ftsZ*‐expressing strains TH‐116Z, TH‐110Z, and TH‐100Z are all haploid cells containing only engineered chromosomes. This also occurred in *E. coli* K‐12 MG1655 (Figure S2a, Supporting Information). The above results suggest that regulating *ftsZ* expression with a weak expression cassette and combining it with chloramphenicol pressure results in polyploid cells. Therefore, regulating *ftsZ* to the appropriate expression level enabled cells to contain both wild‐type and engineered chromosomes.

### 2.2 Confirmation of the Polyploid *E. coli*

**Figure 3:**
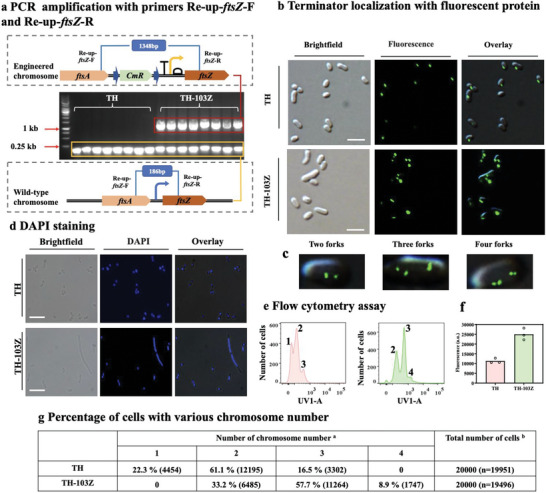
Conformation of the polyploid *E. coli*. a) Chromosome numbers of TH and TH‐103Z cells were analyzed via PCR amplification with primers Re‐up‐*ftsZ*‐F/ Re‐up‐*ftsZ*‐R. b) Terminator imaging of strains TH and TH‐103Z by the GFP‐ParB/*parS* system. c) Two, three, and four forks were formed in TH‐103Z. d) Chromosome imaging of haploid *E. coli* TH and polyploid *E. coli* TH‐103Z using DAPI staining. e,f) Chromosome contents in TH and TH‐103Z were analyzed via DAPI staining with flow cytometry and fluorescence intensity. g) Percentages of cells with various chromosome numbers analyzed via flow cytometry. The size bar is 5 µm. ^a^ Numbers in parentheses indicate cells with corresponding chromosomes. ^b^ n is the number of stained cells examined.

We first validated the polyploidy of *E. coli* TH‐103Z via PCR amplification of the FtsZ expression cassette. Ten isolated colonies were selected from each of *E. coli* TH and TH‐103Z for validation. *E. coli* TH contained 186 bp of the wild‐type chromosome, and *E. coli* TH‐103Z contained both 186 bp of the wild‐type chromosome and 1348 bp of the engineered chromosome (**Figure** **3**a).

Next, we analyzed the chromosome number by locating the terminator in *E. coli* TH‐103Z. Because no Par system exists for plasmid partition in *E. coli*,^[^ ^17^ ^]^ the green fluorescent protein (GFP)‐ParB/*parS* system was used for terminator localization. GFP‐Δ30ParB was assembled by fusing the deleted stop codon of reporter GFP with the ParB that deleted 30 amino acid residues at the N‐terminus.^[^ ^18^ ^]^ To avoid GFP expression throughout the cytoplasm, which leads to poor localization, GFP‐Δ30ParB was integrated into the low‐copy plasmid pCL1920 with the psc101 replication origin. The lac promoter was used to control gene transcription. The *parS* sequence was inserted at ≈20 kb near the chromosome terminator between *gadC* and *gadB*.^[^ ^19^ ^]^ The fused GFP‐Δ30ParB bound to the *parS* sequence to accurately locate the terminator. Wild‐type *E. coli* TH showed one terminator with fluorescent green foci (Figure 3b), whereas two, three or four fluorescent foci were visible in engineered *E. coli* TH‐103Z (Figure 3c). Multiple fluorescent foci were also present in strains TH‐113Z, TH‐109Z, and the model strains MG‐103Z, MG‐113Z, and MG‐109Z (Figure S3, Supporting Information). These results confirmed that we created an artificial polyploid *E. coli* with 2–4 chromosomes.

To calculate the percentage of cells containing 2, 3, or 4 chromosomes in polyploid *E. coli*, the chromosomes were stained with DAPI (Figure 3d), and flow cytometry was used to count the proportions of cells containing different chromosome numbers (Figure 3e). In wild‐type strain TH, 22.3% of cells had one chromosome, 61.1% had two replicating chromosomes, and 16.5% had three chromosomes. In engineered strain TH‐103Z, 33.2% of cells had two chromosomes, 57.7% had three chromosomes, and 8.9% had four chromosomes (Figure 3g). The proportion of DAPI‐stained chromosomes was analyzed by detecting the blue fluorescence intensity, of which, *E. coli* TH‐103Z was ≈2.2 times that of *E. coli* TH (Figure 3f).

### 2.3 Phenotypic Analysis of Polyploid *E. coli*

**Figure 4:**
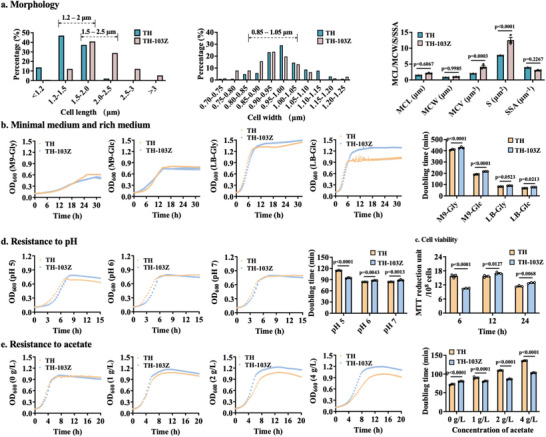
Phenotypic analysis between haploid *E. coli* TH and polyploid *E. coli* TH‐103Z. a) Cell width, length, mean cell length (MCL), mean cell width (MCW), mean cell volume (MCV), surface area (S) and specific surface area (SSA) were calculated from 200 cells imaged by fluorescence microscopy at 100× magnification. b) Growth of strains TH and TH‐103Z cultivated in minimal M9 medium or rich LB medium supplemented with glycerol (Gly) and glucose (Glc) as carbon sources. c) MTT assay for cell viability between TH and TH‐103Z. d) Growth of strains TH and TH‐103Z cultivated in rich LB medium at pH 5, 6, and 7. d, Growth of strains TH and TH‐103Z cultivated in rich LB medium with 0–4 g L^−1^ acetate. All experiments were performed with three (*n* = 3) independent replicates.

The multichromosomal configuration might affect the morphology of the polyploid *E. coli*. Analyzing the shapes of 200 cells showed that the width of both strains was concentrated at 0.85–1.05 µm (**Figure** **4**a). The length of strain TH was concentrated at 1.2–2.0 µm, and that of strain TH‐103z was concentrated at 1.5–2.5 µm (Figure 4a). Compared with those of haploid *E. coli* TH, the mean cell volume and surface area of polyploid TH‐103Z were increased, and the specific surface area was decreased (Figure 4a; Figure S2b, Supporting Information).

Bacterial growth plays a central role in modern microbial physiology and in regulating gene expression.^[^ ^20^ ^]^ Therefore, the phenotype of polyploid *E. coli* TH‐103Z was analyzed via its growth curve and doubling time in the exponential phase,^[^ ^21^ ^]^ including nutrient usage and various stress responses. We first analyzed the growth of polyploid *E. coli* TH‐103Z under different culture conditions. Polyploid *E. coli* TH‐103Z grew slower than did haploid *E. coli* TH with prolonged doubling time under cultivation in minimal M9 medium and rich Luria‐Bertani (LB) medium (Figure 4b). Using glucose as the carbon source in the M9 and LB media increased the doubling times by 13.6% and 13.5%, respectively, for strain TH‐103Z. However, the cell density of strain TH‐103Z was ≈27% higher than that of strain TH when cultured in LB medium. Thus, the polyploid strain TH‐103Z has the properties of slower growth but higher biomass.

pH tolerance is important for maintaining normal bacterial physiological functions.^[^ ^22^ ^]^ The doubling time of polyploid *E. coli* TH‐103Z at pH 5 was 6.5% higher than that at pH 7, but the doubling time of haploid *E. coli* TH increased by 35.9%. TH‐103Z grew better than did TH at pH 5 (Figure 4d). Thus, polyploid *E. coli* TH‐103Z was more resistant to low pH. Acetic acid is the major overflow by‐product in microbial cell factories^[^ ^23^ ^]^ and inhibits cell growth by lowering intracellular pH and disrupting the intracellular anion pool.^[^ ^24^ ^]^ Analysis of the acetate resistance of polyploid *E. coli* TH‐103Z showed that TH‐103Z exhibited more growth than did haploid *E. coli* TH with shortened doubling time after acetate supplementation (Figure 4e). Other phenotypes were also analyzed, including poor resistance to high temperatures and oxidative stress (Figure S4a,b Supporting Information).

Cell viability is an important indicator of cellular metabolic activity.^[^ ^9b^ ^]^ The viability of polyploid *E. coli* was analyzed via MTT assay. Polyploid *E. coli* TH‐103Z had poorer viability in the early‐growth stage, but higher viability in the late‐growth stage (Figure 4c).

### 2.4 Chromosomal Stability and Transcriptomics of Polyploid *E. coli*

**Figure 5:**
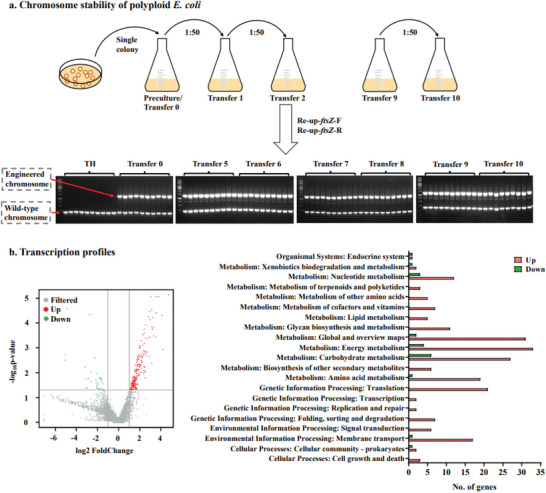
Analysis of chromosome stability and the transcriptome in polyploid *E. coli*. a) Polyploid *E. coli* TH‐103Z was transferred for 10 rounds. The chromosome numbers of TH‐103Z cells from the fifth to tenth rounds were analyzed via PCR amplification with primers Re‐up‐*ftsZ*‐F/Re‐up‐*ftsZ*‐R. b) Significantly differentially expressed genes (*p*<0.05 and |log2 fold change|>1) were screened. The differently expressed genes in the KEGG pathways contained 125 upregulated and 16 downregulated genes.

The stability of the multiple chromosomal configuration is an important property of artificial polyploid *E. coli*. The chromosomal stability of polyploid *E. coli* TH‐103Z was analyzed through continuous transfer every 12 h for ten rounds. Ten single colonies were selected from the fifth to tenth transfers and validated by PCR amplification with primers Re‐up‐*ftsZ*‐F/Re‐up‐*ftsZ*‐R. Until the tenth transfer of ≈62 generations, strain TH‐103Z showed two amplified fragments: 186 bp of the wild‐type chromosome and 1348 bp of the engineered chromosome (**Figure** **5**a and Table S1, Supporting Information). These results demonstrated that polyploid *E. coli* TH‐103Z was highly stable.

Increased chromosome numbers are accompanied by changes in the chromosome epigenome, transcriptome and metabolome.^[^ ^6^ ^]^ Determining the function and significance of chromosome and transcriptome alterations in polyploid cells remains a key challenge.^[^ ^5^ ^]^ We analyzed the differences in gene transcription levels between polyploid *E. coli* TH‐103Z and haploid *E. coli* TH. Transcriptome sequencing was performed on these strains grown to the mid‐exponential phase, and 244 significantly differentially expressed genes (p<0.05 and |log2 fold change|>1) were screened, including 210 upregulated and 34 downregulated genes (Figure 5b, Table S5, Supporting Information). The Kyoto Encyclopedia of Genes and Genomes (KEGG) pathways contained 131 genes, including 125 upregulated and 16 downregulated genes (Table S5, Supporting Information). The upregulated genes were mainly concentrated in major cellular systems, including nucleotide metabolism, global and overview maps, energy metabolism, carbohydrate metabolism, amino acid metabolism, translation, and membrane transport. The transcription levels of abundant division genes, glucose transporter *ptsG*,^[^ ^25^ ^]^ acid adaptation gene islands *gadA*, *gadB*, and *gadC*[26], and acetate‐tolerance genes *murC*, *argA*, *fumB*, *fumC*, and *lcpA* ^[^ ^23b^ ^]^ were upregulated (Table S5, Supporting Information). This may account for the strong tolerance of polyploid *E. coli* to acid and acetate and for its better growth when cultured with glucose as a carbon source.

### 2.5 Efficient Production of L‐threonine by Polyploid *E. coli*

**Figure 6:**
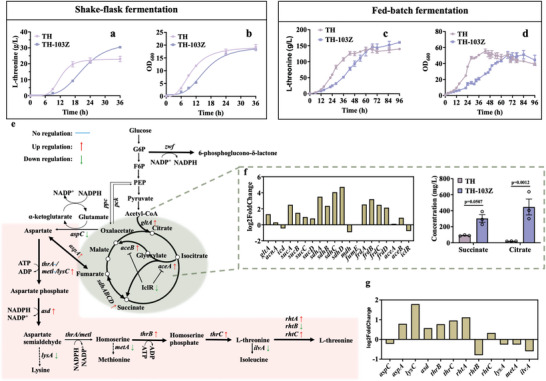
Application of polyploid *E. coli* for L‐threonine production. a,b) Shake‐flask fermentation of haploid strain TH and polyploid strain TH‐103Z. c,d) Fed‐batch fermentation of strains TH and TH‐103Z. e) Metabolic pathway of L‐threonine. f) Transcription levels and metabolites of the tricarboxylic acid cycle. g) Transcription levels of genes related to L‐threonine production pathways. All experiments were performed with three (*n* = 3) independent replicates.

The central metabolic pathway of polyploid *E. coli* was enhanced. Its strong resistance to low pH and acetate and its high viability suggest that it might be able to efficiently produce chemicals. L‐threonine is a high‐value‐added chemical with high global demand, that is currently mainly produced by microbial fermentation. We then analyzed the effect of the increased chromosome number on L‐threonine production with an industrial strain. Strain TH is an engineered L‐threonine‐producing derivative of *E. coli* MG1655, in which the copy number of L‐threonine synthesis genes was increased by rational modification of the MG1655 genome. The transporter protein SstT and the regulatory sequence of the *thrABC* manipulator, *thrL*, were knocked out. Nicotinamide adenine dinucleotide phosphate supply was increased by enhancing *pntAB* expression. For polyploid strain TH‐103Z, the titer, yield, and productivity of L‐threonine after shaken‐flask fermentation reached 30.4 g L^−1^, 0.66 g g^−1^, and 0.844 g L h^−1^, respectively (**Figure** **6**a,b), and in fed‐batch fermentation they reached 160.3 g L^−1^, 0.55 g g^−1^, and 1.66 g L h^−1^, respectively (Figure 6c,d; Figure S5a,c, Supporting Information). These titers were increased by 22.9 g L^−1^ (32.7%) for shaken‐flask fermentation and 139.7 g L^−1^ (14.7%) for fed‐batch fermentation compared with haploid strain TH (Figure 6a,c). In fed‐batch fermentation, strain TH‐103Z produced a 25.9% higher titer than the highest previously reported L‐threonine production in microbial fermentation, with high yield and productivity.^[^ ^27^ ^]^ In fed‐batch fermentation, strain TH‐103Z produced more L‐threonine sedimentation at the bottom of the fermentor than did haploid strain TH (Figure S5b, Supporting Information). For strain TH‐103Z, the succinate and citrate accumulations were increased by 2.3‐fold and 21.4‐fold, respectively, from those of strain TH. Collection of mid‐logarithmic growth phase cells during shaken‐flask fermentation for transcriptomic analysis. The transcription levels were upregulated for nearly all genes in the tricarboxylic acid cycle (TCA cycle), including citrate synthase (*gltA*) and succinic dehydrogenase (*sdhABCD*). The catalytic genes *aceA* and *aceB*, which replenish succinate and malate in the glyoxylic acid cycle, were upregulated, and the negative regulator *iclR* was downregulated (Figure 6e,f). Additionally, genes to related L‐threonine synthesis, including *aspA*, *lysC*, *asd*, *thrB*, *thrC*, transporter *rhtA*, and *rhtC* were upregulated, and competing threonine synthesis genes, including lysine synthesis gene *lysA*, methionine synthesis gene *metA*, and isoleucine synthesis gene *ilvA*, were downregulated (Figure 6g). Thus, polyploid *E. coli* shows great potential for industrial applications.

## 3 Discussion

Using the relationship between replication and division in *E. coli*, we designed a functional polyploid *E. coli* with 2–4 chromosomes by inserting an expression cassette containing *CmR* before the start codon of the essential division gene, *ftsZ*, in a replication fork. The chromosomes of polyploid *E. coli* TH‐103Z were highly stable under chloramphenicol supplementation, which achieved the highest L‐threonine titer reported to date (160.3 g L^−1^). Recently, using the CRISPR strategy under double‐antibiotic screening pressure, Wang et al. constructed a genetically stable diploid *E. coli*.^[^ ^28^ ^]^ In this study, polyploid *E. coli* was constructed by weakly expressing *ftsZ* and adding chloramphenicol to the culture medium. Due to changes in division of the polyploid *E. coli* in our study, the cells may have been inconsistent with conventional *E. coli* replication and division.^[^ ^29^ ^]^ This resulted in cells with unfixed numbers of 2–4 chromosomes, possibly because of unstable cell division in multiple chromosomal cells.^[^ ^2^ ^]^ Although the reason for the inconsistent chromosome numbers in our polyploid cells is unclear, the one‐step design provides a new method for developing polyploid cells from haploid cells. Cell division is regulated by dozens of proteins, including FtsZ, that coordinate with each other to control the separation of daughter cells.^[^ ^30^ ^]^ We believe that, in addition to *ftsZ*, this method is suitable for other essential division genes in *E. coli*, such as *ftsB*, *ftsL*, and *zipA*. Prokaryotes reproduce by binary fission, in which the chromosome is replicated bidirectionally starting at the origin, progressing to the terminators. The divisome is then recruited through the Z‐ring formed by FtsZ, such as in *C. glutamicum* and *Bacillus subtilis*, to synthesize new cell walls and a septum, eventually generating newborn daughter cells.^[^ ^30^, ^31^ ^]^ Therefore, this strategy can also be applied to other prokaryotes.

Evolutionary polyploid cells are considered to be the end of evolution, usually accompanied by changes in morphology, physiology and metabolism mediated by gene expression and epigenetic remodeling.^[^ ^2^, ^6^ ^]^ Transcriptomic analysis showed upregulation of replication, transcription, and translation genes. The red fluorescent protein (RFP) expression level of polyploid *E. coli* TH‐103Z was significantly higher than that of haploid *E. coli* TH, indicating that the translational efficiency of polyploid *E. coli* might be increased (Figure S6, Supporting Information). A recent study indicated that diploid *E. coli* had stronger viability than did haploid *E. coli* under UV radiation.^[^ ^28^ ^]^ We obtained consistent conclusions. For example, strain TH‐103Z had strong tolerance to low pH and acetate but poor tolerance to heat shock and oxidative stress. In addition, high stability of the chromosomes was demonstrated in both this study and that of Wang et al. ^[^ ^32^ ^]^ Although both the polyploid *E. coli* in this study and the diploid *E. coli* constructed by Wang et al. exhibited slower growth than haploid *E. coli* when cultured in LB medium, the polyploid *E. coli* had higher biomass relative to the haploid *E. coli*. Cell robustness is an important feature in resistance to unfavorable culture environments in microbial production.^[^ ^33^ ^]^ During microbial fermentation, large amounts of acetic acid are produced, and its accumulation inhibits cell growth and gene expression. Many studies have improved production of chemicals by enhancing the tolerance of microbes to acetic acid.^[^ ^34^ ^]^ The level of gene expression is another important bottleneck in production of chemicals. Overexpression of pathway genes increases the carbon flux and the productivity.^[^ ^35^ ^]^ The polyploid *E. coli* cells created in this study were highly tolerant of low pH and acetate, and had high gene expression efficiency. In addition, polyploid *E. coli* had significantly increased transcription levels of major biological pathways, such as the TCA cycle, and pathways for amino acid metabolism and energy metabolism. Thus, polyploid *E. coli* may also be applied to enhance the synthesis of other bio‐based chemicals. Therefore, we conclude that compared with haploid *E. coli*, polyploid *E. coli* offers the advantage of stress resistance but also has some tolerance defects. In conclusion, we designed an easy and convenient method for constructing polyploid *E. coli* with 2–4 chromosomes. This polyploid *E. coli* shows great potential for industrial production.

## 4 Experimental Section

### Reagents

4′,6‐diamidine‐2′‐phenylindole dihydrochloride (DAPI) and L‐threonine standard were purchased from Sigma‐Aldrich (St. Louis, MO, USA). Glucose, glycerol, hydrochloric acid, hydrogen peroxide (30% aqueous solution), and acetic acid were purchased from Sinopharm Chemical Reagent Beijing Co. Ltd. (Shanghai, China). Phanta HS Super‐Fidelity DNA Polymerase was purchased from Vazyme Biotech (Nanjing, China). High‐performance liquid chromatography (HPLC)‐grade acetonitrile (75‐05‐8) was obtained from Tedia (ACN, Tedia Company, Inc., Fairfield, OH, USA). Triethylamine (CAS, 121‐44‐8) and phenyl isothiocyanate (CAS, 103‐72‐0) were purchased from Aladdine. Chloramphenicol was purchased from Sangon Biotech Co., Ltd. (Shanghai, China). Size Separation Master Kit was purchased from ProteinSimple (San Jose, CA). HRP Goat Anti‐Mouse IgG (H+L) (Catalog NO: AS003) was purchased from ABclonal Technology Co.,Ltd.

### Strains, Plasmids, Primers and Growth Conditions

Tables S2 and S3 (Supporting Information) list all strains, plasmids and primers used. *E. coli* DH5α was used for plasmid reconstruction. Strain TH was used in the polyploid experiments and was provided by the Fufeng Group (http://www.fufeng‐group.com/). 16S sequence alignment results showed that strain TH belonged to *E. coli*, showing 100% identity with *E. coli* K‐12 MG1655 (Table S6, Supporting Information). Plasmids pTKRED and pKD3 were used in genome editing. LB medium containing 5 g L^−1^ yeast extract, 10 g L^−1^ tryptone, and 10 g L^−1^ NaCl was used for plasmid construction, terminator localization and phenotypic analysis. M9 medium consisting of 17.1 g L^−1^ Na_2_PO_4_∙12 H_2_O, 3 g L^−1^ KH_2_PO_4_, 0.5 g L^−1^ NaCl, 1 g L^−1^ NH_4_Cl, 1 mM MgSO_4_∙7H_2_O, 0.1 mM CaCl_2_, and 20 g L^−1^ glucose was used for phenotypic analysis. The shake‐flask fermentation medium consisted of 15 g L^−1^ (NH4)_2_SO_4_, 2 g L^−1^ KH_2_PO_4_, 1 g L MgSO_4_∙7H_2_O, 2 g L^−1^ yeast extract, 0.02 g L^−1^ FeSO_4_, and 40 g L^−1^ glucose. CaCO_3_ (20 g L^−1^) was added to adjust the pH. The fed‐batch fermentation medium consisted of 20 g L^−1^ (NH4)_2_SO_4_, 2 g L^−1^ KH_2_PO_4_, 3 g L^−1^ yeast extract, 2 g L^−1^ MgSO_4_∙7H_2_O, 5 mg L^−1^ FeSO_4_, 5 mg L^−1^ MnSO_4_·4H_2_O, 20 g L^−1^ glucose, and 0.5 g L^−1^ glycine betaine.

### Plasmid and Strain Construction

The characterization plasmids pCL‐100RFP, pCL‐110RFP, pCL‐116RFP, pCL‐109RFP, pCL‐113RFP, and pCL‐103RFP were constructed by assembling the amplified backbone from pCL1920 with reporter gene *rfp* and J23100/J23110/J23116‐B0034 or J23103/J23113/J23109‐B0033 using the Gibson assembly method.^[^ ^36^ ^]^ For terminator localization, the prophage of bacteriophage P1 *parB* was synthesized by General Biotech (Anhui) Co., Ltd. (http://www.generalbiol.com/). The N‐terminal‐truncated ParB was fused with stop codon‐deleted GFP, resulting in GFP‐Δ30 ParB. GFP‐Δ30 ParB was then inserted into pCL1920 to construct pCL‐GFP‐Δ30 ParB. Table S4 (Supporting Information) shows the GFP‐Δ30 ParB sequence. Plasmid pE‐RFP was constructed by inserting the expression cassette J23100‐B0034‐*rfp* into pETDuet‐1.

To control *ftsZ* expression, different promoter strengths, including J23100, J23110, J23116, J23109, J23113, and J23103 (derived from iGEM: http://parts.igem.org/Promoters/Catalog/Constitutive) and ribosome‐binding sites, including B0033 and B0034 (derived from iGEM: http://parts.igem.org/Ribosome_Binding_Sites) were inserted before the start codon of *ftsZ* in the strain TH chromosome using homologous recombination,^[^ ^37^ ^]^ resulting in engineered strains TH‐100Z, TH‐110Z, TH‐116Z, TH‐109Z, TH‐113Z, and TH‐103Z. Terminator B1006 (derived from iGEM: http://parts.igem.org/Terminators) was inserted before the promoters to avoid effects of the upstream gene. For terminator location, TH‐*parS* and TH‐103Z‐*parS* were constructed by inserting the P1 *parS* site between *gadB* and *gadC*,^[^ ^19a^ ^]^ 20 kb from the *dif* site at the chromosome terminus of strains TH and TH‐103Z. Table S4 (Supporting Information) shows the P1 *parS* sequence. Strains TH‐*parS*‐ParB and TH‐103Z‐*parS*‐ParB were constructed by transferring plasmid pCL‐GFP‐Δ30 ParB. Plasmid pE‐RFP was transformed into strains TH and TH‐103Z to generate TH‐RFP and TH‐103Z‐RFP, respectively. Table S3 (Supporting Information) lists all primers used for strain and plasmid construction.

### Characterization of the FtsZ Expression Cassette Intensity

To compare the FtsZ intensities in strains TH‐100Z, TH‐110Z, TH‐116Z, TH‐109Z, TH‐113Z, and TH‐103Z, plasmids pCL‐100RFP, pCL‐110RFP, pCL‐116RFP, pCL‐109RFP, pCL‐113RFP, and pCL‐103RFP were transformed into strain TH for characterization. All strains used were incubated for 24 h at 37 °C in 24‐well plates containing 1.5 mL LB medium. The fluorescence intensity was measured through excitation at 590 nm and emission at 645 nm using a multidetection microplate reader (Synergy HT, BioTek, Winooski, VT, USA). The fluorescence intensity values were normalized to cell density (OD_600_).

### Western Blotting

Cells were collected by centrifugation at 12 000 rpm for 10 min at 4 °C after 6 h of incubation in LB medium at 37 °C and 220 rpm. The cells were resuspended in 10 mL of phosphate buffer solution (pH 7.2) and 100 µL of protease inhibitor (0.1 M benzylsulfonyl fluoride dissolved in isopropanol) was added. Cells were disrupted by ultrasonication for 20 min (cycles of 4 s on and 6 s off). The supernatant was collected by centrifugation at 12 000 rpm for 20 min at 4 °C. Western immunoblotting was performed on an automated Simple Wes System (ProteinSimple, CA, USA).^[^ ^38^ ^]^ Data were analyzed using Compass software (version 6.2, ProteinSimple).

### Terminator Location

Plasmid pCL‐GFP‐Δ30 ParB was transferred into strains TH‐*parS*, TH‐103Z‐*parS*, TH‐113Z‐*parS*, TH‐109Z‐*parS*, MG‐*parS*, MG‐103Z‐*parS*, MG‐113Z‐*parS*, MG‐109Z‐*parS* to generate strains TH‐*parS*‐ParB, TH‐103Z‐*parS*‐ParB, TH‐113Z‐*parS*‐ParB, TH‐109Z‐*parS*‐ParB, MG‐*parS*‐ParB, MG‐103Z‐*parS*‐ParB, MG‐113Z‐*parS*‐ParB, MG‐109Z‐*parS*‐ParB, respectively. The strains were cultivated at 37 °C in 10 mL LB medium. Spectinomycin dihydrochloride pentahydrate was added during cultivation to maintain GFP‐Δ30 ParB expression. Cells were collected for microscopy after reaching the exponential phase.

### Fluorescence Microscopy

Culture medium (1 mL) was centrifuged at 12 000 rpm for 1 min to remove the supernatant. The collected cells were resuspended by adding 100 µL fresh LB medium. Next, 5 µL of cell resuspension solution was transferred to polylysine‐coated glass slides. The cells were covered with a coverslip and immobilized by pressing the coverslip. Cells were visualized using a Nikon Eclipse 80i microscope (Nikon Corp. Tokyo, Japan) at 100× magnification.

### Cell Size Calculation

The parameters of strains TH and TH‐103Z were calculated using 200 individual cells. Cell length and width were measured through microscopic imaging at 100× magnification using a Nikon Eclipse 80i microscope. The volume and surface area were calculated using the equations:^[^ ^39^ ^]^

(1) $$S\;=\;2\pi R\left(L-2R\right)+4\pi R^{2}$$

(2) $$V\;=\pi R^{2}\;\left(L-2R\right)+4/3\pi R^{3}$$

In these equations, *R* is mean cell width, *L* is mean cell length, *V* is mean cell volume, and *S* is surface area. Specific surface area was calculated by dividing *S* by *V*.

### Field Emission Scanning Electron Microscope (FESEM) Assay

Cultivated cells (1 mL) were collected by centrifugation in 2000 r min^−1^ for 2 min. The cells were washed three times with phosphate buffer at pH 7.2, then collected by centrifugation and immobilized with 2.5% glutaraldehyde at 4 °C for 3 h. The immobilized cells were washed again three times. Cells were then dehydrated through a gradient concentration of ethanol (30%, 50%, 70%, 80%, 90%, and 100%). Cells were dried to critical point with an automated dryer (Leica EM CPD300; Leica Microsystems GmbH, Wetzlar, Germany) and coated with gold sputter. Cell images were obtained via FESEM (Quanta 250 FEG; FEI Ltd., Brno, Czech Republic).

### DAPI Staining

Strains were harvested by centrifugation at 12 000 r min^−1^ for 2 min. The collected cells were washed three times with phosphate buffer at pH 7.2. After removing the supernatant, 1 mL of 3.7% formaldehyde was added, and the cells were left to stand for 30 min at 4 °C. The fixed cells were then washed three times. To increase cell permeability, the fixed cells were treated with 5% Triton X‐100 for 30 min at 4 °C. Cells were then gently washed three times. The supernatant was completely removed, and 10 µL of 5 mg L^−1^ DAPI was added and resuspended. The mixture was placed in the dark for 10 min for chromosome staining. The stained cells were washed three times to remove the excess DAPI. The fluorescence intensity of the stained chromosomes was measured through excitation at 364 nm and emission at 454 nm using a multidetection microplate reader (Synergy HT, BioTek, Winooski, VT, USA). The fluorescence values were normalized to cell density (OD_600_).

### Flow Cytometry

Strains were cultured in 10 mL LB medium at 37 °C for 12 h. For TH‐103Z cultivation, 34 µg mL^−1^ chloramphenicol was added to maintain the polyploidy. Cells were stained with DAPI and diluted to OD <0.1 using phosphate buffer. Samples were filtered with a 300‐mesh filter prior to flow cytometry. Fluorescence intensity of the cells was analyzed using an imaging flow cytometer (ImageStreamXMarkII, Amnis/Merck). A total of 20 000 cells per sample were collected for analysis. Blue fluorescence was detected in the UV1 channel. Data were analyzed using flow cytometry analysis software (Flowjo VX10).

### Phenotype Experiment

The phenotypes of strains TH and TH‐103Z were analyzed by measuring cell growth and doubling time in the exponential phase.^[^ ^21^ ^]^ Single colonies of strains TH and TH‐103Z were grown in 10 mL LB medium at 37 °C for 12 h. The seeds were transferred to a 48‐well microassay plate containing 700 µL LB or M9 medium with 2% (v/v) inoculation. For temperature‐affected analysis, cells were cultivated individually at 30, 37, 42 or 50 °C. LB and M9 media supplemented with 20 g L^−1^ glycerol or 20 g L^−1^ glucose were used to study the influence of different culture media and carbon sources between haploid strain TH and polyploid strain TH‐103Z. To analyze acid resistance, cells were cultivated in LB medium at pH 5, 6, 7 adjusted with hydrochloric acid.^[^ ^40^ ^]^ To analyze the oxidative stress sensitivity, cells were cultivated in LB medium at pH 7 in H_2_O_2_ at 0, 7, 14, 21, 28, or 35 mM.^[^ ^41^ ^]^ To analyze the acetate‐stress sensitivity, cells were cultivated in LB with acetate at 0, 1, 2, 4, or 6 g L^−1^ titrated to neutral pH with KOH.^[^ ^23b^ ^]^ To cultivate strain TH‐103Z, 34 µg mL^−1^ chloramphenicol was added to maintain the polyploidy. Doubling time (min) of the cells was calculated during the exponential phase with at least five continuous time points using the following equation:

(3) $$Doubling\;time=\left(t_{j}-t_{i}\right)/\log2\;\left(\frac{C_{j}}{C_{i}}\right)$$

where *t_j_* and *t_i_* represent the two time points. *C_j_* and *C_i_* represent the two OD_600_ values corresponding to *t_j_* and *t_i_*.

### Gene Expression

Fluorescence protein was used to analyzed gene expression.^[^ ^42^ ^]^ To compare gene expression levels between strains TH and TH‐103Z, RFP expression between strains TH and TH‐103Z was detected under the control of constitutive promoter J23100 and ribosome‐binding site B0034. Plasmid pE‐RFP was transferred into TH and TH‐103Z to generate TH‐RFP and TH‐103Z‐RFP, respectively. Single colonies of strains TH‐RFP and TH‐103Z‐RFP were grown in 10 mL LB medium at 37 °C for 12 h. The seeds were then transferred to a 48‐well microassay plate containing 700 µL LB medium and M9 medium supplemented with glycerol or glucose at 37 °C for 36 h. The RFP expression level was also studied in LB medium without glycerol or glucose. RFP was detected through excitation at 590 nm and emission at 645 nm. During the characterization process, strain TH‐RFP was supplemented with 100 µg mL^−1^ ampicillin sodium, and strain TH‐103Z‐RFP was supplemented with 100 µg mL^−1^ ampicillin sodium and 34 µg mL^−1^ chloramphenicol.

### MTT Assay

The MTT assay was performed as previously described.^[^ ^43^ ^]^ The principle of this assay was based on succinate dehydrogenase reduction of the added MTT to water‐insoluble formazan. The light absorption values were measured at 490 nm after the dimethyl sulfoxide was dissolved.^[^ ^9^, ^43^ ^]^ Formazan accumulation was proportional to the number of viable cells.^[^ ^9^, ^43^ ^]^ Strains TH and TH‐103Z were cultivated in LB medium or fermentation medium at 37 °C. Cells were harvested at various growth stages and diluted to OD_600_ 0.1. Diluted bacterial culture (200 µL) was transferred to a 1.5‐mL centrifuge tube, then 100 µL 5 mg mL^−1^ MTT was added and mixed. The mixture was incubated at 37 °C for 4 h, then the pellet was collected by centrifuging at 12 000 rpm for 2 min. Next, 200 µL dimethyl sulfoxide was added to dissolve the pellet, and 100 µL dissolved solution was transferred to a 96‐well microassay plate for MTT assay at 490 nm using a multidetection microplate reader (Synergy HT, BioTek, Winooski, VT, USA). The MTT reduction unit was calculated as previously described.^[^ ^43b^ ^]^

### Transcriptome Analysis

Single colonies of strains TH and TH‐103Z were cultivated in 10 mL LB medium at 37 °C for 12 h. The seeds were transferred to a 300‐mL shake flask containing 20 mL shake‐flask fermentation medium with 2% (v/v) inoculation at 37 °C. Chloramphenicol (34 µg mL^−1^) was added to the strain TH‐103Z cultivation to maintain polyploidy. TH and TH‐103Z cells were collected in the mid‐exponential phase for transcriptome analysis, which was conducted at OE Biotech Co., Ltd. (Shanghai, China). Total RNA was extracted using the mirVana miRNA isolation kit (Ambion). RNA integrity was evaluated using the Agilent 2100 Bioanalyzer (Agilent Technologies, Santa Clara, CA, USA). Samples with RNA integrity numbers ≥7 were subsequently analyzed. Libraries were constructed using TruSeq Stranded Total RNA with Ribo‐Zero Gold per the manufacturer's instructions. These libraries were then sequenced on the Illumina sequencing platform (HiSeq™ 2500 or other platform), and 150 bp/125 bp paired‐end reads were generated. Three independent biological replicates of strains TH and TH‐103Z were performed.

### L‐threonine Fermentation and Chromosomal Stability of Polyploid E. coli

Shake‐flask fermentation was performed as previously described.^[^ ^44^ ^]^ Briefly, single colonies of TH and TH‐103Z were cultivated in LB medium at 37 °C for 12 h. The precultured seeds were transferred to a 300‐mL shake flask containing 20 mL fermentation medium with 1% (v/v) inoculation at 220 rpm at 37 °C for 36 h. Chloramphenicol (34 µg mL^−1^) was added to the strain TH‐103Z fermentation.

For fed‐batch fermentation, the preculture was transferred into fresh LB medium 10% (v/v) inoculation as secondary seeds. Then, 10% (v/v) of the secondary seed culture was inoculated into a 7.5‐L bioreactor (Infors HT, Bottmingen, Switzerland) containing 4 L of fed‐batch fermentation medium. Chloramphenicol (34 µg mL^−1^) was added to the strain TH‐103Z fermentation. The pH was adjusted to 7.0 with ammonium hydroxide. Glucose was added as a carbon source starting at 40 g L^−1^, then supplemented to 20–40 g L^−1^ while ensuring that it did not drop below 10 g L^−1^. The fermentation temperature was controlled at 37 °C. The dissolved oxygen level was controlled at ≥30% by adjusting the agitation speed and airflow rate. The L‐threonine titer was detected as previously described.^[^ ^44^ ^]^ Succinate and citrate were measured via HPLC (Shimadzu, Japan) equipped with a refractive index detector (RID‐10A; Shimadzu, Japan) and an Aminex HPX‐87H ion exclusion column (Bio‐Rad, USA) as described previously.^[^ ^45^ ^]^

To analyze the chromosomal stability of polyploid *E. coli*, haploid *E. coli* TH and polyploid *E. coli* TH‐103Z were continuously transferred every 12 h under shake‐flask fermentation with a 2% (v/v) inoculation ratio. Strain TH‐103Z was processed by adding 34 µg mL^−1^ chloramphenicol. Ten rounds of transfers were conducted, corresponding to approximately 62 generations. From rounds 5–10, ten colonies per transfer were analyzed via PCR using primers Re‐up‐ftsZ‐F and Re‐down‐ftsZ‐R. The generation was calculated using the following formula:^[^ ^46^ ^]^

(4) $$\mathrm{Generation}=\log2\;\left(\frac{N_{f}}{N_{0}}\right)$$

*N_f_*: final population size; *N_0_*: initial population size

### Statistical Analysis

Results were presented as the mean and standard error of the mean (± SEM). Differences between means were evaluated using two‐way analysis of variance in GraphPad Prism 9 Project software. *p* < 0.05 was considered statistically significant.

## Conflict of Interest

The authors declare no conflict of interest.

## Author Contributions

S.W. investigate, conceptualized, and wrote the original draft of this article. X.C. performed the experiment of terminator localization. Experiments of scanning electron micrographs and phenotypic analysis were conducted by Xin Jin. F.G. performed DAPI staining and flow cytometry assay. W.J. conducted the chromosome stability and L‐threonine fermentation. Q.Q. and Q.L. performed the conceptualization, funding acquisition, resource management, project administration, supervision, and wrote, reviewed and edited. All authors have read and agreed to the published version of the manuscript.

## Supporting information

Supporting Information

Supplemental Table 5

Supplemental Table 6

## Data Availability

The data that support the findings of this study are available in the supplementary material of this article.
