# Supplementary material for: Creating Polyploid Escherichia Coli and Its Application in Efficient L‐Threonine Production
Source: Adv Sci (Weinh). 2023 Sep 25;10(31):2302417. doi: 10.1002/advs.202302417 (PMC10625114; doi:10.1002/advs.202302417)
Supplement: Supplementary file 1 — Supporting Information [file ADVS-10-2302417-s002.pdf]

ADVANCED SCIENCE
Open Access

Supporting Information

for *Adv. Sci.*, DOI 10.1002/advs.202302417

Creating Polyploid *Escherichia Coli* and Its Application in Efficient L-Threonine Production

Sumeng Wang, Xuanmu Chen, Xin Jin, Fei Gu, Wei Jiang, Qingsheng Qi and Quanfeng Liang**

Supporting Information

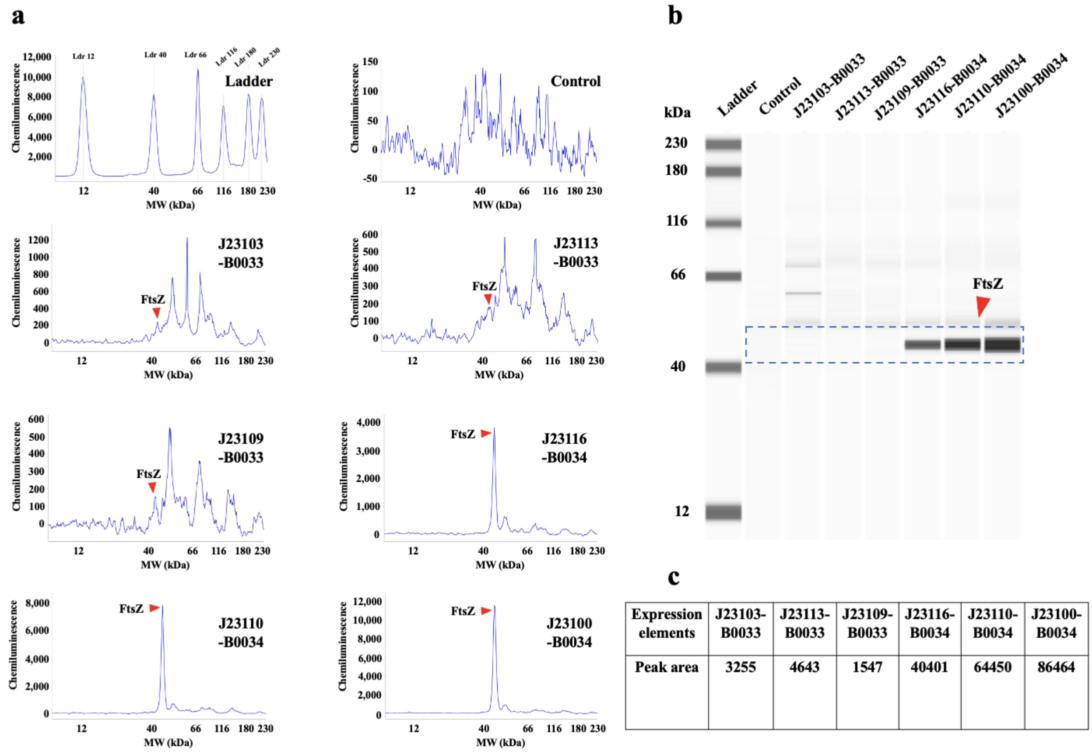

c

Expression elements	J23103-B0033	J23113-B0033	J23109-B0033	J23116-B0034	J23110-B0034	J23100-B0034
Peak area	3255	4643	1547	40401	64450	86464

Figure S1. Expression level of FtsZ controlled by expression elements J23103-B0033, J23113-B0033, J23109-B0033, J23116-B0034, J23110-B0034, J23100-B0034 were detected by capillary western blot. Strain MG1655 without FtsZ expression as the control. a, results are shown as electropherograms. b, results are shown as gel-like image view. c, results are shown as peak area.

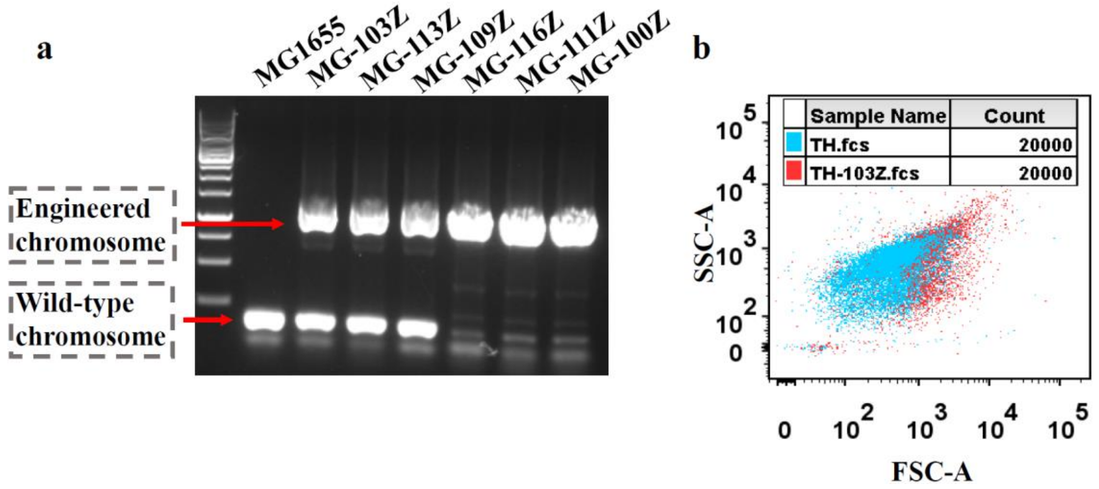

Figure S2. Analysis of chromosome number, morphology. a, Chromosome number of *E. coli* K-12 MG1655 with different intensities of FtsZ were analyzed through the PCR

amplification with primers (Re-up-*ftsZ*-F/ Re-up-*ftsZ*-R) in upstream and downstream of the integrated expression cassette Cm-terminator-promoter-RBS. b, Comparation of the size between TH and TH-103Z by flow cytometry with total of 20,000 cells of each sample.

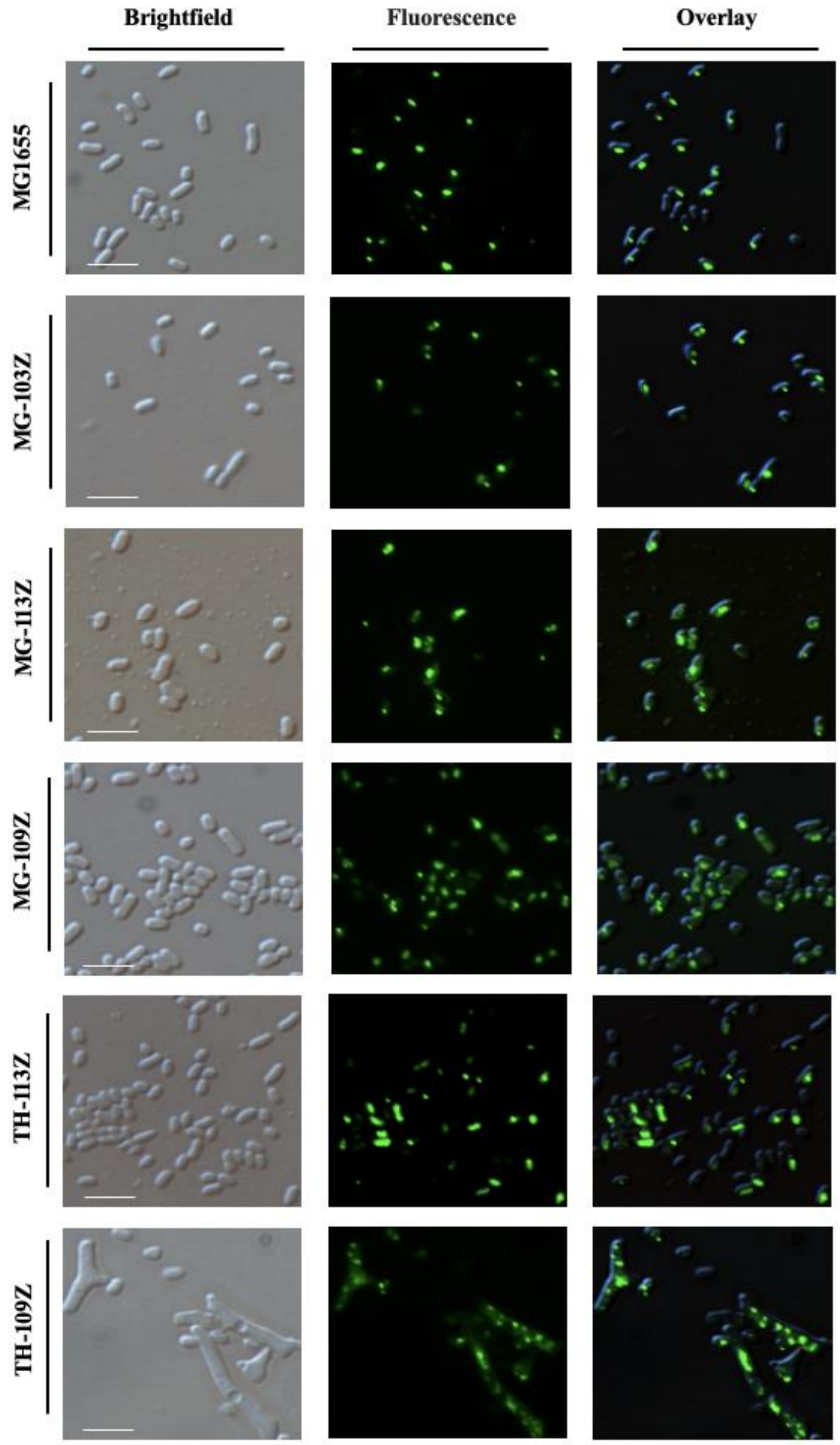

Figure S3. Terminator imaging of strains MG1655, MG-103Z, MG-113Z, MG-109Z, TH-113Z, TH-109Z by the GFP-ParB/*parS* system. The size bar is 5 μm.

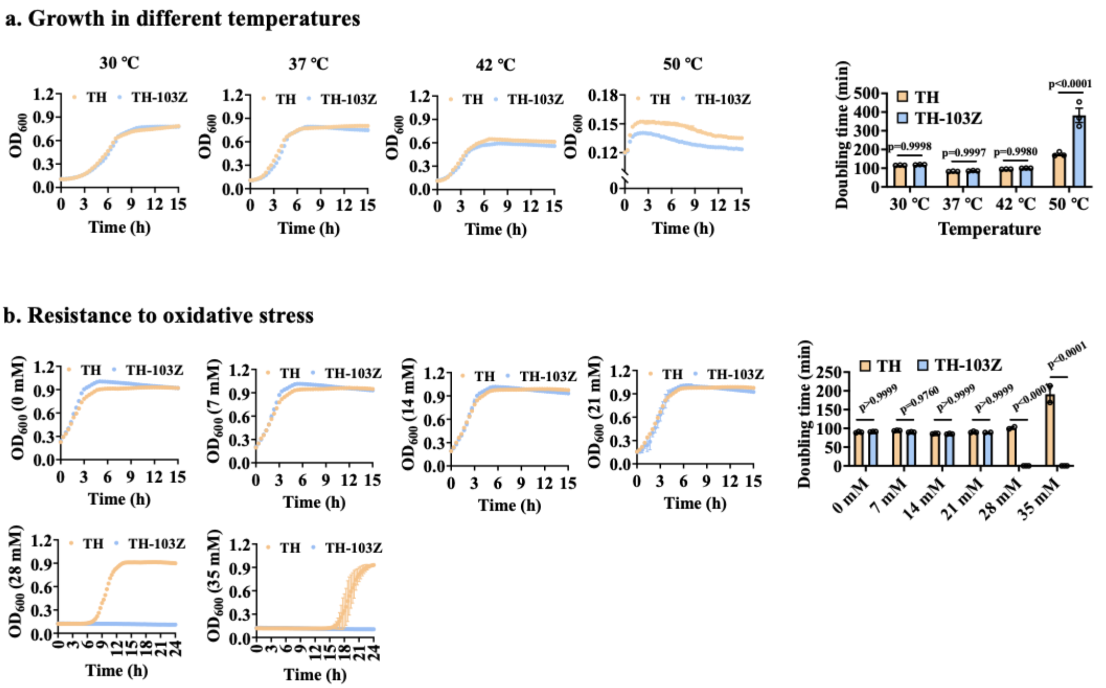

Figure S4. Analyzing the cell growth at different temperature and oxidative stress in polyploid *E. coli*. a, Growth of haploid *E. coli* TH and polyploid *E. coli* TH-103Z were cultivated in LB medium at 30 °C, 37 °C, 42 °C, 50 °C. b, Growth of haploid *E. coli* TH and polyploid *E. coli* TH-103Z were cultivated in rich medium LB at different concentration of H_2O_2. All results were performed with three (n = 3) independent replicates.

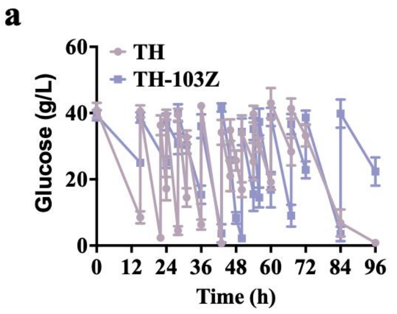

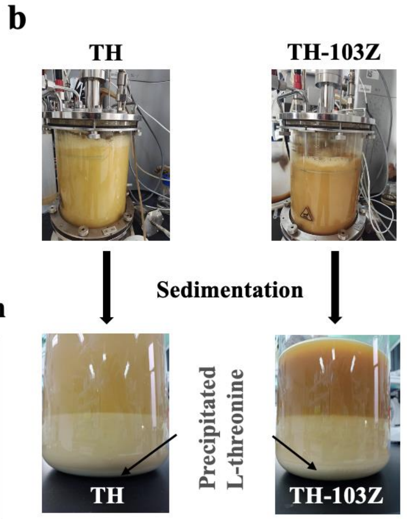

c L-threonine production in fed-batch fermentation

	L-threonine (g/L)	Consumed glucose (g/L)	Yield (g/g)	Productivity (g/L/h)
TH	139.7	333.0	0.41	1.45
TH-103Z	160.3	287.3	0.55	1.66

Figure S5. Application of polyploid *E. coli* for L-threonine production in fed-batch fermentation. a, Glucose consumption of haploid *E. coli* TH and polyploid *E. coli* TH-

103Z in fed-batch fermentation. b, Precipitated L-threonine in fed-batch fermentation of strains TH and TH-103Z. c, L-threonine titer, consumed glucose, L-threonine yield and productivity of strains TH and TH-103Z in fed-batch fermentation. All experiments were performed with three (n = 3) independent replicates.

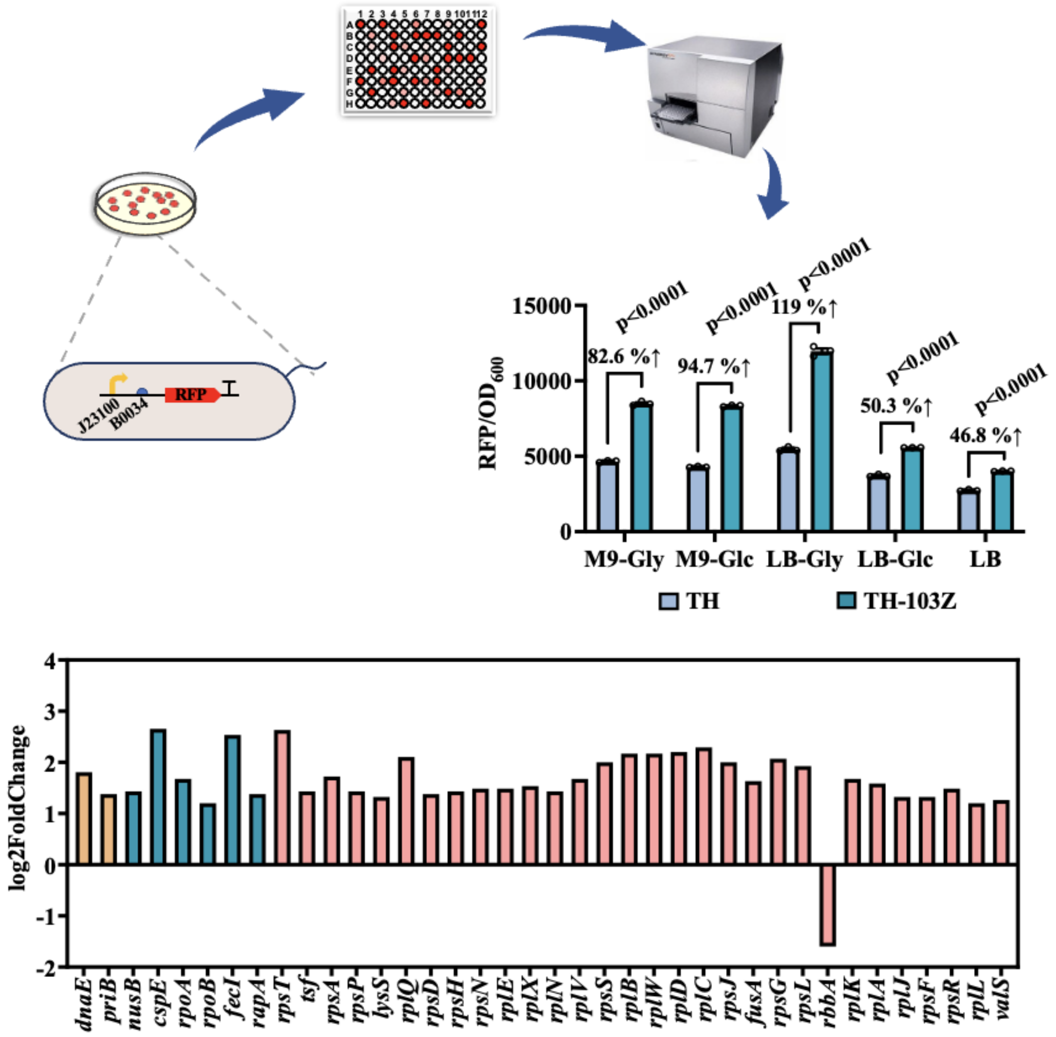

Figure S6. Assay of the protein expression levels of TH and TH-103Z by the characterization of RFP/OD_{600}. Inserting the fused red fluorescent protein (RFP) with constitutive promoter J23100 and RBS B0034 into plasmid pETDUet-1. Transcription levels of genes in replication, transcription and translation. All results were performed with three (n = 3) independent replicates.

Table S1. Generations of transferred haploid *E. coli* TH and polyploid *E. coli* TH-103Z

	Transfer 1	Transfer 2	Transfer 3	Transfer 4	Transfer 5	Transfer 6	Transfer 7	Transfer 8	Transfer 9	Transfer 10

TH	9.84624	15.44693	21.19149	26.87006	33.31268	39.01897	44.56534	50.0881	55.89841	61.42205
TH-103Z	8.42873	14.06962	19.93476	25.97586	31.89493	37.62114	43.34244	48.91542	54.96682	61.69914

Table S2. Plasmids and strains used in this study.

Plasmids	Description	Source
pCL1920	rep_{pSC101} Spc^R P_{lac}	[1]
pTKRED	rep_{pSC101} Spc^R ParaBAD Rep101(Ts) lac UV5 promoter RecA Gam Beta Exo	[2]
pKD3	R6K γ ori Cm^R FRT AmpR	[3]
pCL-100RFP	rep_{pSC101} Spc^R P_{J23100} B0034 - *rfp*	This study
pCL-110RFP	rep_{pSC101} Spc^R P_{J23110} B0034 - *rfp*	This study
pCL-116RFP	rep_{pSC101} Spc^R P_{J23116} B0034 - *rfp*	This study
pCL-109RFP	rep_{pSC101} Spc^R P_{J23109} B0033 - *rfp*	
pCL-113RFP	rep_{pSC101} Spc^R P_{J23113} B0033 - *rfp*	This study
pCL-103RFP	rep_{pSC101} Spc^R P_{J23103} B0033 - *rfp*	This study
pCL-GFP-Δ30 ParB	rep_{pSC101} Spc^R P lac lac operator GFP - Δ30 ParB	This study
pE-100RFP	ColE1 ori Amp^R P_{J23100} B0034 - *rfp*	This study
pCL-103-FtsZ	reppSC101 SpcR P_{J23103} B0033 –*ftsZ*-his tag	This study
pCL-113-FtsZ	reppSC101 SpcR P_{J23113} B0033 –*ftsZ*-his tag	This study
pCL-109-FtsZ	reppSC101 SpcR P_{J23109} B0033 –*ftsZ*-his tag	This study
pCL-116-FtsZ	reppSC101 SpcR P_{J23116} B0034 –*ftsZ*-his tag	This study
pCL-110-FtsZ	reppSC101 SpcR P_{J23110} B0034 –*ftsZ*-his tag	This study
pCL-100-FtsZ	reppSC101 SpcR P_{J23100} B0034 –*ftsZ*-his tag	This study
Strains		
E. coli DH5α	F^- supE44 ΔlacU169 (*ϕ80 lacZ ΔM15*) *hsdR*17 *recA*1 *endA*1 *gyrA96* *thi*-1 *relA*1	Invitrogen
E. coli K-12 MG1655	K12 F- *lambda- ilvG- rfb-50 rph-1*	Invitrogen
TH	L-threonine producing *E. coli* strain	FuFeng Group
TH-*parS*	P1 *parS* was inserted between *gadB* and *gadC* in the genome of the TH strain	This study
TH-RFP	TH carrying pE-100RFP	This study

TH-103Z-RFP	TH-103Z carrying pE-100RFP	This study
TH-103Z-*parS*	P1 *parS* was inserted between *gadB* and *gadC* in the genome of the TH-103Z strain	This study
TH-113Z-*parS*	P1 *parS* was inserted between *gadB* and *gadC* in the genome of the TH-113Z strain	This study
TH-109Z-*parS*	P1 *parS* was inserted between *gadB* and *gadC* in the genome of the TH-109Z strain	This study
TH-*parS*-ParB	TH-*parS* carrying pCL-GFP-Δ30 ParB	This study
TH-103Z-*parS*-ParB	TH-103Z-*parS* carrying pCL-GFP-Δ30 ParB	This study
TH-113Z-*parS*-ParB	TH-113Z-*parS* carrying pCL-GFP-Δ30 ParB	This study
TH-109Z-*parS*-ParB	TH-109Z-*parS* carrying pCL-GFP-Δ30 ParB	This study
MG-*parS*	P1 *parS* was inserted between *gadB* and *gadC* in the genome of the MG1655 strain	This study
MG-103Z-*parS*	P1 *parS* was inserted between *gadB* and *gadC* in the genome of the MG-103Z strain	This study
MG-113Z-*parS*	P1 *parS* was inserted between *gadB* and *gadC* in the genome of the MG-113Z strain	This study
MG-109Z-*parS*	P1 *parS* was inserted between *gadB* and *gadC* in the genome of the MG-109Z strain	This study
MG-*parS*-ParB	MG-*parS* carrying pCL-GFP-Δ30 ParB	This study
MG-103Z-*parS*-ParB	MG-103Z-*parS* carrying pCL-GFP-Δ30 ParB	This study
MG-113Z-*parS*-ParB	MG-113Z-*parS* carrying pCL-GFP-Δ30 ParB	This study
MG-109Z-*parS*-ParB	MG-109Z-*parS* carrying pCL-GFP-Δ30 ParB	This study
TH-103Z	Derived from TH by inserting DNA sequence FRT-*CmR*-FRT-B1006-J23103-B0033 before start codon of *ftsZ*	This study
TH-113Z	Derived from TH by inserting DNA sequence FRT-*CmR*-FRT-B1006-J23113-B0033 before start codon of *ftsZ*	This study
TH-109Z	Derived from TH by inserting DNA	This study

	sequence FRT-*CmR*-FRT-B1006-J23109-B0033 before start codon of *ftsZ*	
TH-116Z	Derived from TH by inserting DNA sequence FRT-*CmR*-FRT-B1006-J23116-B0034 before start codon of *ftsZ*	This study
TH-110Z	Derived from TH by inserting DNA sequence FRT-*CmR*-FRT-B1006-J23110-B0034 before start codon of *ftsZ*	This study
TH-100Z	Derived from TH by inserting DNA sequence FRT-*CmR*-FRT-B1006-J23100-B0034 before start codon of *ftsZ*	This study
MG-103Z	Derived from MG1655 by inserting DNA sequence FRT-*CmR*-FRT-B1006-J23103-B0033 before start codon of *ftsZ*	This study
MG-113Z	Derived from MG1655 by inserting DNA sequence FRT-*CmR*-FRT-B1006-J23113-B0033 before start codon of *ftsZ*	This study
MG-109Z	Derived from MG1655 by inserting DNA sequence FRT-*CmR*-FRT-B1006-J23109-B0033 before start codon of *ftsZ*	This study
MG-116Z	Derived from MG1655 by inserting DNA sequence FRT-*CmR*-FRT-B1006-J23116-B0034 before start codon of *ftsZ*	This study
MG-110Z	Derived from MG1655 by inserting DNA sequence FRT-*CmR*-FRT-B1006-J23110-B0034 before start codon of *ftsZ*	This study
MG-100Z	Derived from MG1655 by inserting DNA sequence FRT-*CmR*-FRT-B1006-J23100-B0034 before start codon of *ftsZ*	This study
MG-103Z-his	MG1655 carrying pCL-103-FtsZ	This study
MG-113Z-his	MG1655 carrying pCL-113-FtsZ	This study
MG-109Z-his	MG1655 carrying pCL-109-FtsZ	This study
MG-116Z-his	MG1655 carrying pCL-116-FtsZ	This study
MG-110Z-his	MG1655 carrying pCL-110-FtsZ	This study
MG-100Z-his	MG1655 carrying pCL-100-FtsZ	This study

Table S3. Primers used in this study.

Primer name	sequences (5’-3’)
pCL-100RFP reconstruction	
CL-10034-R1	gagctagcactgtacctaggactgagctagccgtcaagcctggggtgcctaatgagtga
CL-10034-R2	ctagtatttctcctctttctctagagctagcactgtacctaggactgagct
34RFP-F	tctagagaaagaggagaaatactagatggcttcctccgaagacgt

34RFP-R	ttaagcgtagttttcgtcgtttgc
pCL-110RFP reconstruction	
CL-11034-R1	gctagcattgtacctaggactgagctagccgtaaagcctggggtgcctaatgagtga
CL-11034-R2	ctagtatttctcctctttctctagagctagcattgtacctaggactgagc
34RFP-F	tctagagaaagaggagaaatactagatggcttcctccgaagacgt
34RFP-R	ttaagcgtagttttcgtcgtttgc
pCL-116RFP reconstruction	
CL-11634-R1	gagctagcatagtccctaggactgagctagctgtcaagcctggggtgcctaatgagtga
CL-11634-R2	ctagtatttctcctctttctctagagctagcatagtccctaggactgagctagct
34RFP-F	tctagagaaagaggagaaatactagatggcttcctccgaagacgt
34RFP-R	ttaagcgtagttttcgtcgtttgc
pCL-109RFP reconstruction	
CL-10933-R1	gctagcacagtccctaggactgagctagctgtaaagcctggggtgcctaatgagtga
CL-10933-R2	ctagtagtcctgtgtgactctagagctagcacagtccctaggactgagctagct
33RFP-F	ctctagagtcacacaggactactagatggcttcctccgaagacgt
34RFP-R	ttaagcgtagttttcgtcgtttgc
pCL-113RFP reconstruction	
CL-11333-R1	gctagcataatccctaggactgagctagccatcaggcctggggtgcctaatgagtga
CL-11333-R2	ctagtagtcctgtgtgactctagagctagcataatccctaggactgagct
33RFP-F	ctctagagtcacacaggactactagatggcttcctccgaagacgt
34RFP-R	ttaagcgtagttttcgtcgtttgc
pCL-103RFP reconstruction	
CL-10333-R1	gctagcataatccctaggactgagctagctatcaggcctggggtgcctaatgagtga
CL-11333-R2	ctagtagtcctgtgtgactctagagctagcataatccctaggactgagct
33RFP-F	ctctagagtcacacaggactactagatggcttcctccgaagacgt
34RFP-R	ttaagcgtagttttcgtcgtttgc
pCL-GFP-Δ30	
ParB reconstruction	
B-cl-ParB-R	tccagtgaaaagttcttctcctttacgcattggcgtaatcatggtcatagctgt
B-cl-ParB-F	tattctgcgtaaaagtctggataaaaaaccgaaaccgtaaagcttgcatgcctgcaggt
GFP-F	atgcgtaaaggagaagaacttttcac
GFP-R	tttgtatagttcatccatgccatgtg
ParB-F	attacacatggcatggatgaactatacaaagttgaacaggtgtttaaactgagtaccg
ParB-R	ttacggtttcggtttttatccagac
pE-100RFP reconstruction	
B-pE-F	gctgcagcaaacgacgaaaactacgcttaatggcagatctcaattggatatcggc

B-pE-R	atcgagatcgatctcgatcctct
100RFP-F1	atggcttcctccgaagacgt
100RFP-F2	gtacagtgctagctctagagaaagaggagaaatactagatggcttcctccgaagacgt
100RFP-F3	ccggcgtagaggatcgagatcgatctcgatttgacggctagctcagtcctaggtacagtgct agctctagagaaagagg
100RFP-R	ttaagcgtagttttcgtcgt
pCL-100-FtsZ reconstruction	
cl-100Z-F	ttgacggctagctcagtcctag
cl-100-BR	cactgtacctaggactgagctagccgtcaagcctggggtgcctaatgagt
cl-BF	caagctgatcatcaccatcatcaccactaaagcttgcatgcctgcaggtc
cl-100Z-R	ttagtggtgatgatggtgatgatcagcttgcttacgcaggaat
pCL-110-FtsZ reconstruction	
cl-110Z-F	tttacggctagctcagtcctaggt
cl-110-BR	gctagcattgtacctaggactgagctagccgtaaagcctggggtgcctaatgagtga
cl-BF	caagctgatcatcaccatcatcaccactaaagcttgcatgcctgcaggtc
cl-100Z-R	ttagtggtgatgatggtgatgatcagcttgcttacgcaggaat
pCL-116-FtsZ reconstruction	
cl-116Z-F	ttgacagctagctcagtcctag
cl-116-BR	gagctagcatagtccctaggactgagctagctgtcaagcctggggtgcctaatgagtga
cl-BF	caagctgatcatcaccatcatcaccactaaagcttgcatgcctgcaggtc
cl-100Z-R	ttagtggtgatgatggtgatgatcagcttgcttacgcaggaat
pCL-109-FtsZ reconstruction	
cl-109Z-F	tttacagctagctcagtcctaggga
cl-109-BR	gctagcacagtccctaggactgagctagctgtaaagcctggggtgcctaatgagtga
cl-BF	caagctgatcatcaccatcatcaccactaaagcttgcatgcctgcaggtc
cl-100Z-R	ttagtggtgatgatggtgatgatcagcttgcttacgcaggaat
pCL-113-FtsZ reconstruction	
cl-113Z-F	ctgatggctagctcagtcctagg
cl-113-BR	cataatccctaggactgagctagccatcaggcctggggtgcctaatgagtga
cl-BF	caagctgatcatcaccatcatcaccactaaagcttgcatgcctgcaggtc
cl-100Z-R	ttagtggtgatgatggtgatgatcagcttgcttacgcaggaat
pCL-103-FtsZ reconstruction	
cl-103Z-F	ctgatagctagctcagtcctagggat
cl-103-BR	gctagcataatccctaggactgagctagctatcaggcctggggtgcctaatgagtga
cl-BF	caagctgatcatcaccatcatcaccactaaagcttgcatgcctgcaggtc
cl-100Z-R	ttagtggtgatgatggtgatgatcagcttgcttacgcaggaat
Re-up-*ftsZ*-F	gcgactcaatagttggctgc
Re-down-*ftsZ*-	cgcaccatgtgttcaacagca

R	
test-CmR-F	ctggcgattcaggttcatcatg
test-ftsZ-R	cggacgagtaatcagttcagcgat
up-ftsZ-F	gagtctgcaacgtcagacact
up-ftsZ-R	tatggaccatggctaattcccatgtcagccagtttctctccgatttgtgcctg
pKD3-F	ggctgacatgggaattagccat
pKD3-R	gcattacacgtcttgagcgattg
1006-R	aaaaaaaaccccgccctgtcaggggcggggttttttttgcattacacgtcttgagcgattgtg ta
1006-33-R	cataatccctaggactgagctagctatcagaaaaaaaaccccgccctgtc
103Z-F	ctgatagctagctcagtcctagggattatgctagctctagagtcacacaggactactag
down-ftsZ-33F	tctagagtcacacaggactactagatgtttgaaccaatggaacttacc
down-ftsZ-R	cagaactttcagcagtttgtcg
113Z-F	ctgatggctagctcagtcctagggattatgctagctctagagtcacacaggactactag
113Z-R	cataatccctaggactgagctagccatcagaaaaaaaaccccgccctgtc
109Z-F	tttacagctagctcagtcctagggactgtgctagctctagagtcacacaggactactag
109Z-R	cacagtccctaggactgagctagctgtaaaaaaaaaaaccccgccctgtc
down-ftsZ-34F	tctagagaaagaggagaaatactagatgtttgaaccaatggaacttacc
116Z-F	agctagctcagtcctagggactatgctagctctagagaaagaggagaaatactag
116Z-R	catagtccctaggactgagctagctgtcaaaaaaaaaaccccgccctgtc
110Z-F	ggctagctcagtcctaggtacaatgctagctctagagaaagaggagaaatactag
110Z-R	cattgtacctaggactgagctagccgtaaaaaaaaaaaccccgccctgtc
100Z-F	tgacggctagctcagtcctaggtacagtgctagctctagagaaagaggagaaatactag
100Z-R	cactgtacctaggactgagctagccgtcaaaaaaaaaaccccgccctgtc

Table S4. Sequences used in this study.

Name	Sequences
B1006	aaaaaaaaaccccgcccctgacagggcggggttttttt
B0033	tctagagtcacacaggactactag
B0034	tctagagaaagaggagaaatactag
J23100	ttgacggctagctcagtcctaggtacagtgctagc
J23110	tttacggctagctcagtcctaggtacaatgctagc
J23116	ttgacagctagctcagtcctagggactatgctagc
J23109	tttacagctagctcagtcctagggactgtgctagc
J23113	ctgatggctagctcagtcctagggattatgctagc
J23103	ctgatagctagctcagtcctagggattatgctagc

P1 *parS*	gtgaaatcgtggcgatttcaccttgaaattttagagtaatttactttaaaaacagtcagttaatagt gaaatttgaatggcga
GFP-Δ30 ParB	atgcgtaaaggagaagaacttttcactggagttgtcccaattcttgttgaattagatggtgatgtta atgggcacaaattttctgtcagtggagagggtgaaggtgatgcaacatacggaaaacttaccc ttaaatttatttgcactactggaaaactacctgttccatggccaacacttgtcactactttcggttat ggtgttcaatgctttgcgagatacccagatcatatgaaacagcatgactttttcaagagtgccat gcccgaaggttatgtacaggaaagaactatatttttcaaagatgacgggaactacaagacacg tgctgaagtcaagtttgaaggtgatacccttgttaatagaatcgagttaaaaggtattgattttaaa gaagatggaaacattcttggacacaaattggaatacaactataactcacacaatgtatacatcat ggcagacaaacaaaagaatggaatcaaagttaacttcaaaattagacacaacattgaagatgg aagcgttcaactagcagaccattatcaacaaaatactccaattggcgatggccctgtccttttac cagacaaccattacctgtccacacaatctgccctttcgaaagatcccaacgaaaagagagacc acatggtccttcttgagtttgtaacagctgctgggattacacatggcatggatgaactatacaaa gttgaacaggtgtttaaactgagtaccggccgtcaggccacctttattgaagaagttattccgcc gaatcaggttgaaagcgatacctttgtggatcagcataataatggccgtgatcaggcaagtctg accccgaaaagtctgaaaagcattcgtagcaccattaagcatcagcagttttatccggccattg gcgtgcgccgtgccaccggaaaaattgaaattctggatggcagtcgtcgtcgcgccagtgcc attctggaaaatgtgggcctgcgcgttctggttaccgatcaggaaattagcgtgcaggaagca cagaatctggcaaaagatgttcagaccgccctgcaacatagtattcgtgaaattggcctgcgc ctgatgcgcatgaaaaatgatggtatgagtcagaaagatattgccgcaaaagaaggcctgagt caggccaaagtgacccgtgcactgcaagccgcaagcgccccggaagaactggtggcactg tttccggtgcagagtgaactgacctttagtgattataaaccctgtgtgcagttggcgatgaaat gggtaataagaatctggaatttgatcagctgattcagaatattagcccggaaattaatgatattct gagcattgaagagatggccgaagatgaagtgaaaaataagattctgcgcctgattaccaaag aagccagtctgctgaccgataaaggcagtaaagataaaagcgtggttaccgaactgtggaaa tttgaagataaagatcgctttgcacgcaaacgtgtgaaaggtcgtgcctttagttatgagtttaat cgcctgagtaaagaactgcaagaagaactggatcgcatgattggccatattctgcgtaaaagt ctggataaaaaaccgaaaccgtaa

Table S5. Transcriptome analysis of haploid strain TH and polyploid strain TH-103Z. Total of 244 significantly differentially expressed genes ($p<0.05$ and |log2 fold change|>1), including 125 upregulated and 16 downregulated genes in KEGG (Kyoto Encyclopedia of Genes and Genomes) pathway.

Table S6. 16S rRNA genes in the genome of strain TH and the sequence alignment.

References

[1] C. G. Lerner, M. Inouye, *Nucleic acids research* **1990**, *18* (15), 4631, https://doi.org/10.1093/nar/18.15.4631.
[2] T. E. Kuhlman, E. C. Cox, *Nucleic acids research* **2010**, *38* (6), e92, https://doi.org/10.1093/nar/gkp1193.
[3] K. A. Datsenko, B. L. Wanner, *Proceedings of the National Academy of Sciences of the United States of America* **2000**, *97* (12), 6640, https://doi.org/10.1073/pnas.120163297.
